# One-Pot Synthesis of a Robust Crosslinker-Free Thermo-Reversible Conducting Hydrogel Electrode for Epidermal Electronics

**DOI:** 10.1021/acsami.3c10663

**Published:** 2024-01-12

**Authors:** Nazmi B. Alsaafeen, Sarah S. Bawazir, Kishore K. Jena, Aibobek Seitak, Bushara Fatma, Charalampos Pitsalidis, Ahsan Khandoker, Anna-Maria Pappa

**Affiliations:** † Department of Biomedical Engineering, Khalifa University, Abu Dhabi 127788, UAE; ‡ Department of Physics, Khalifa University, Abu Dhabi 127788, UAE; § Healthcare Engineering Innovation Center, Khalifa University, Abu Dhabi 127788, UAE; ∥ Center for Catalysis and Separation, Khalifa University, Abu Dhabi 127788, UAE

**Keywords:** ECG monitoring, conducting hydrogels, wearable electronics, graphene, MXene, PEDOT:PSS, conducting polymers

## Abstract

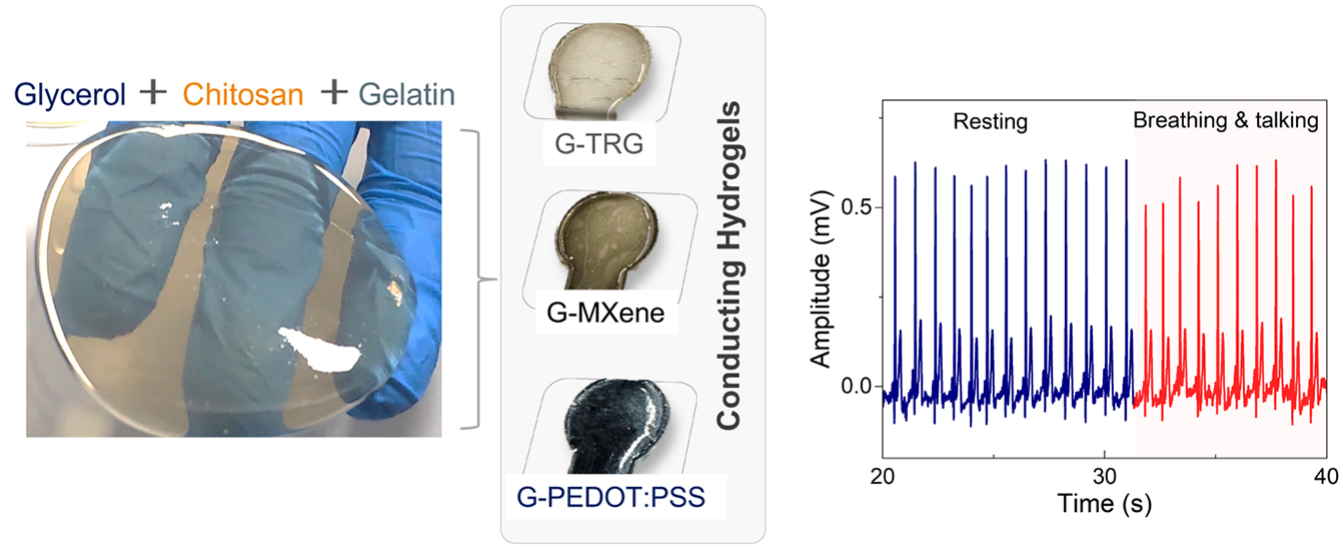

Traditional epidermal electrodes, typically made of silver/silver chloride (Ag/AgCl), have been widely used in various applications, including electrophysiological recordings and biosignal monitoring. However, they present limitations due to inherent material mismatches with the skin. This often results in high interface impedance, discomfort, and potential skin irritation, particularly during prolonged use or for individuals with sensitive skin. While various tissue-mimicking materials have been explored, their mechanical advantages often come at the expense of conductivity, resulting in low-quality recordings. We herein report the facile fabrication of conducting and stretchable hydrogels using a “one-pot” method. This approach involves the synthesis of a natural hydrogel, termed *Golde*, composed of abundant and eco-friendly components, including gelatin, chitosan, and glycerol. To enhance the conductivity of the hydrogel, various conducting materials, such as poly(3,4-ethylenedioxythiophene) polystyrenesulfonate (PEDOT:PSS), thermally reduced graphene (TRG), and MXene, are introduced. The resulting conducting hydrogels exhibit remarkable robustness, do not require crosslinkers, and possess a unique thermo-reversible property, simplifying the fabrication process and ensuring enhanced long-term stability. Moreover, their fabrication is sustainable, as it employs environmentally friendly materials and processes while retaining their skin-friendly characteristics. The resulting hydrogel electrodes were tested for electrocardiogram (ECG) signal acquisition and outperformed commercial electrodes even when implemented in an all-flexible electrode setup simply using copper tape, owing to their inherent adhesiveness.

## Introduction

Epidermal electronics have emerged as non-invasive, compact, and ergonomic tools to monitor an individual’s health status while resting and during dynamic conditions such as sweating or exercising.^1^ Commercial electrophysiology devices are typically metal electrodes such as silver/silver chloride (Ag/AgCl) that, although highly conductive, are flat and rigid and hence do not adequately conform to human skin. This mismatch often leads to skin irritation, electrode degradation, and sweat-induced failure, all of which hamper signal quality in the long term.^2,3^ Furthermore, those electrodes have a finite shelf life^4^ due to the gradual conversion of Ag to AgCl over time, even during storage, severely impacting the stability and reliability of the electrodes.

Conducting hydrogels combine the water absorption and retention properties of hydrogels with the conductivity typically provided by conducting polymers or carbon-based materials, making them highly suitable for advanced and comfortable wearable devices.^5^ These hydrogels possess inherent adhesiveness and superior skin conformability and exhibit mixed conduction (both ionic and electronic), along with remarkable mechanical properties, surpassing the performance of conventional metal electrodes. Thus, they serve as a more biomimetic alternative for sensing, stimulating, and modulating electrical activity in biological tissues.^6^ Furthermore, conducting hydrogels conform to the skin, reducing the gap between the electrode and the skin and minimizing skin–electrode contact impedance, hence enhancing the signal-to-noise ratio (SNR) of electrophysiological measurements.^7,8^

However, several aspects need to be addressed for clinical and commercial translation to fully realize the potential of conducting hydrogels. The main challenge is optimizing the electrical conductivity such that it is comparable to that of metal electrodes while also ensuring long-term stability and biocompatibility. To render hydrogels electrically conductive, standard methods include doping them with ionic liquids, metallic nanoparticles, carbon-based conductive fillers, or conducting polymers.^9−11^ Furthermore, developing scalable fabrication methods that keep the cost of the materials and processes as low as possible is another critical requirement.^12^

Hydrogels can be synthesized using various methods, such as polymerization,^13^ crosslinking,^11,14^ solution mixing,^11,15^ radiation,^16,22^ etc. They can be made from synthetic materials, including poly(vinyl alcohol), polyacrylamide, and polyethylene glycol,^1,5,13^ natural materials, including gelatin, chitosan, and alginate,^17−21^ or hybrids.^17,22,23^ While synthetic hydrogels might seem superior in terms of mechanical properties, they often suffer in terms of biocompatibility, biodegradability, and sustainability. Natural hydrogels are arguably more desirable, especially given their high resemblance to the skin, low toxicity, sustainability, and facile processing.^17,24^ However, they still need the addition of chemical crosslinkers, which are often toxic, as they tend to degrade in water and suffer in terms of mechanical robustness and stability.

Gelatin, a form of hydrolyzed collagen, is a protein that constitutes the majority of human skin composition. Being biocompatible, biodegradable, cheap, and relatively highly abundant, gelatin has been widely used in several biological applications. For example, in wearables, it facilitates the formation of polyelectrolyte complexes (PECs) and adherence to the skin, as it possesses a variety of functional groups such as amino (R–NH_2_), hydroxyl (R–OH), and carboxyl (R–COOH) groups.^21^ Several studies have hence focused their attention on gelatin-based hydrogel electronics, endowing the hydrogel with electrical conductivity, for example, by sandwiching it with two-dimensional (2D) materials such as polypyrrole and reduced graphene oxide.^17^ Chitosan, the second-most abundant bioadhesive polysaccharide^26^ similar to gelatin, is both biocompatible and biodegradable, exhibits ionic conductivity, and has the ability to form intermolecular interactions such as PECs due to its functional groups.^18,19,27^ Finally, glycerol, a triol natural plasticizer, has also been employed in several hydrogel electrodes.^5,11,28^ For example, increasing the ratio of bound water molecules against free water molecules using its hydroxyl groups has been shown to promote hydrogen bonding,^28^ thereby reducing the gelation time and making the resulting hydrogel more elastic and robust^11^ with nondrying and antifreezing behavior.^5,16,28^

**Figure 1:**
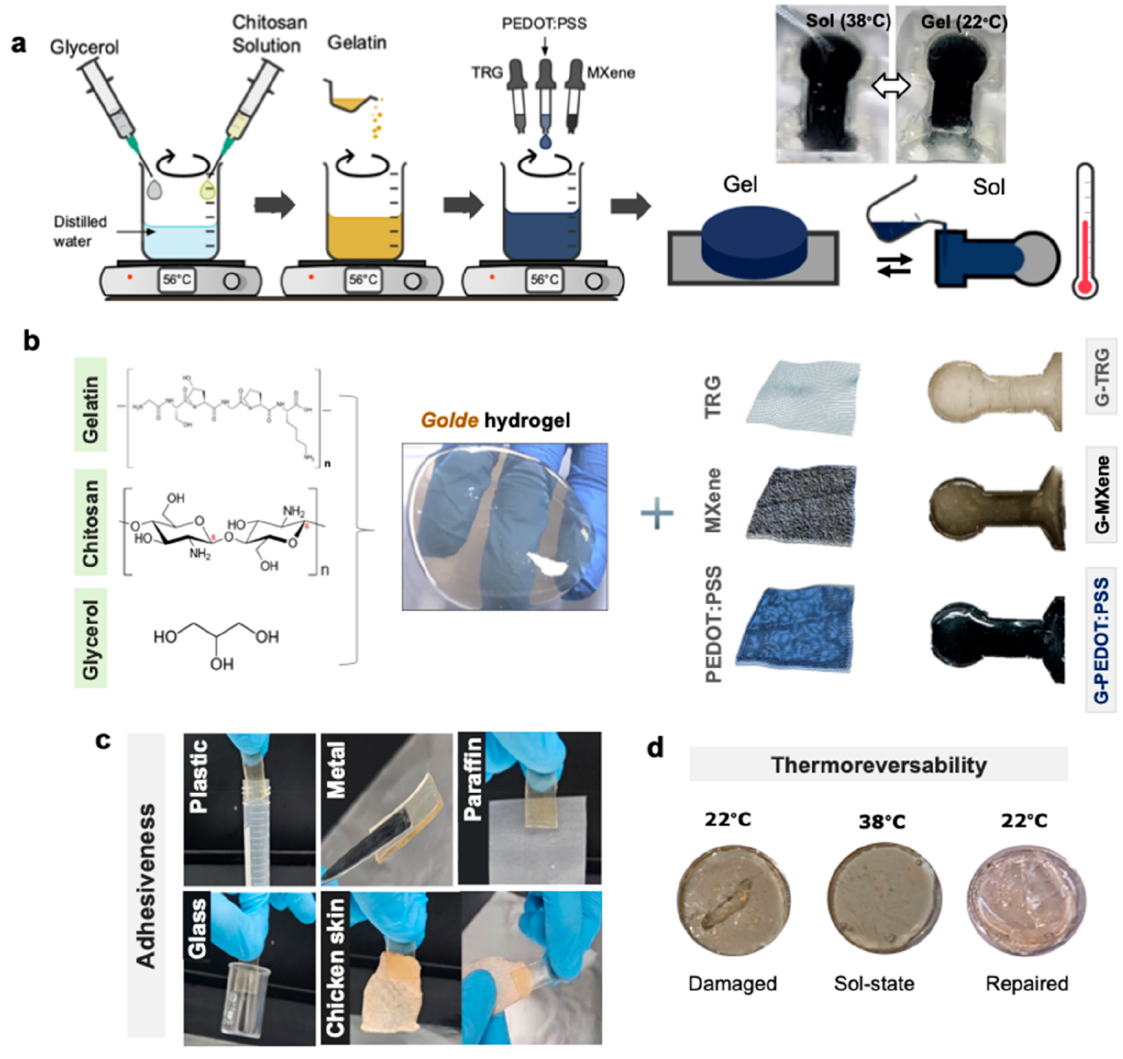
Hydrogel preparation. (a) Schematic showing one-pot synthesis method of the conducting hydrogels. (b) Chemical structures of the main components comprising the conducting hydrogel electrodes, and a photograph of the actual hydrogels (dimensions: 1.7 cm circle diameter and 1 cm × 1.3 cm rectangle) (c) Adhesiveness demonstration of the hydrogels with various materials including plastic, metal, paraffin, glass, and skin. (d) Photographs of the resulting hydrogels being repaired after cracking, showcasing their thermo-reversibility at the corresponding temperatures.

In this work, we combine gelatin, chitosan, and glycerol through a one-pot fabrication approach to benefit from their synergistic properties, as shown in Figure 1a. *Golde*, the resulting hydrogel depicted in Figure 1b, bypasses the use of typical crosslinkers such as dimethyl sulfoxide (DMSO) or other potentially toxic chemical agents, as it relies on a high density of interpolymeric interactions including electrostatic interactions, polyelectrolyte complexes, hydrogen bonding, and chain entanglements. The combination of chitosan, a cationic polysaccharide, and gelatin, an amphoteric protein at certain pH, enables the formation of electrostatically entangled PECs that form polymeric architectures with relatively high tensile strength for a natural hydrogel.^20^*Golde* demonstrates several attractive features: it is robust, highly stretchable, inherently adhesive, and thermo-reversible. Thermo-reversibility facilitates the repair of the hydrogel upon drying or cracking, which makes it reusable (Figure 1a,d). Further, this allows for flexibility and versatility in its preparation, as it can be reprocessed any time or can be used as a viscous ink for 3D printing. To enhance its conducting properties, we investigated the addition of three solution-processable conducting materials, as shown in Figure 1b: MXene (Ti_3_C_2_T_*x*_), thermally reduced graphene (TRG), and poly(3,4-ethylenedioxythiophene) polystyrenesulfonate (PEDOT:PSS). The composition of the hydrogel variants was optimized to yield homogeneous dispersions of the conductive materials in the hydrogel solution for optimal robustness and conductivity. We show that all three resulting hydrogel electrodes are robust, each having mechanical strength that outperforms that of state-of-the-art natural and hybrid-based hydrogels (see Table S1 for comparisons), and that they can conform to the skin with the potential for high-quality electrocardiogram (ECG) recording. Owing to the inherent adhesiveness of the hydrogel on a variety of surfaces including skin, as demonstrated in Figure 1c, an all-flexible electrode was used to record a Lead I ECG by directly interfacing a healthy 24-year-old male. Our work presents a facile and eco-friendly solution for designing sustainable, wearable materials for epidermal electronics and electrophysiological recordings that demonstrate superior performance compared to commercial electrodes.

## Results and Discussion

### Material Selection

We combined three natural materials to create a robust base hydrogel, termed *Golde*, which does not require the use of chemical crosslinkers and features tissue-like mechanical properties and thermo-reversibility, which are important for its synthesis and post-synthesis modification.^11^ Typically, mechanical stability and integrity are imparted via crosslinking, i.e., the formation of chemical or physical bonds between the polymer chains within a hydrogel network. Crosslinking creates a three-dimensional network that prevents the dissolution or disintegration of the hydrogel in an aqueous environment, allowing the hydrogel to maintain its shape, strength, and functionality and to withstand mechanical forces. Here, chitosan creates a PEC with gelatin, resulting in a tough natural hydrogel with a relatively high tensile strength without the addition of any external crosslinking agent.^29^ In addition, no mold or fouling was observed in the hydrogel samples after three months of storage in ambient room conditions, possibly attributed to chitosan’s antimicrobial properties.^26^ Furthermore, the integration of glycerol was found to improve the hydrogel’s elasticity while inhibiting its dehydration by acting as a plasticizer and a humectant.^11^ To render our hydrogel furtherly conductive and lower the electrode impedance, we blended our base material with three different conducting materials: poly(3,4-ethylenedioxythiophene) polystyrenesulfonate (PEDOT:PSS), MXene, and thermally reduced graphene (TRG). All three materials were processed from water suspensions and were mixed directly with a *Golde* solution (as shown in Figure 1a,b). PEDOT:PSS, a conducting polymer that has been widely used in wearable electronics,^30^ typically interacts with other charged polymers, in this case chitosan, creating complexes that may improve the overall hydrogel properties.^31^ MXene is a composite of a metallic carbonitride, in our case titanium carbide, written in the following notation Ti_3_C_2_T_*x*_, where T represents the surface functional group (−OH, −F, −O). MXene (Ti_3_C_2_T_*x*_) exhibits metallic conductivity as well as tunable surface functional groups and a negatively charged hydrophilic surface, enabling strong interactions with polymer networks, such as electrostatic interactions and hydrogen bonding.^33^ TRG features a large surface area, and in addition to improving the conductivity of the hydrogel, its (nano)structure could act as mechanical reinforcement for the hydrogel, providing stability and conducting paths through interpolymeric interactions.^17^

### Surface Chemistry and Morphology

**Figure 2:**
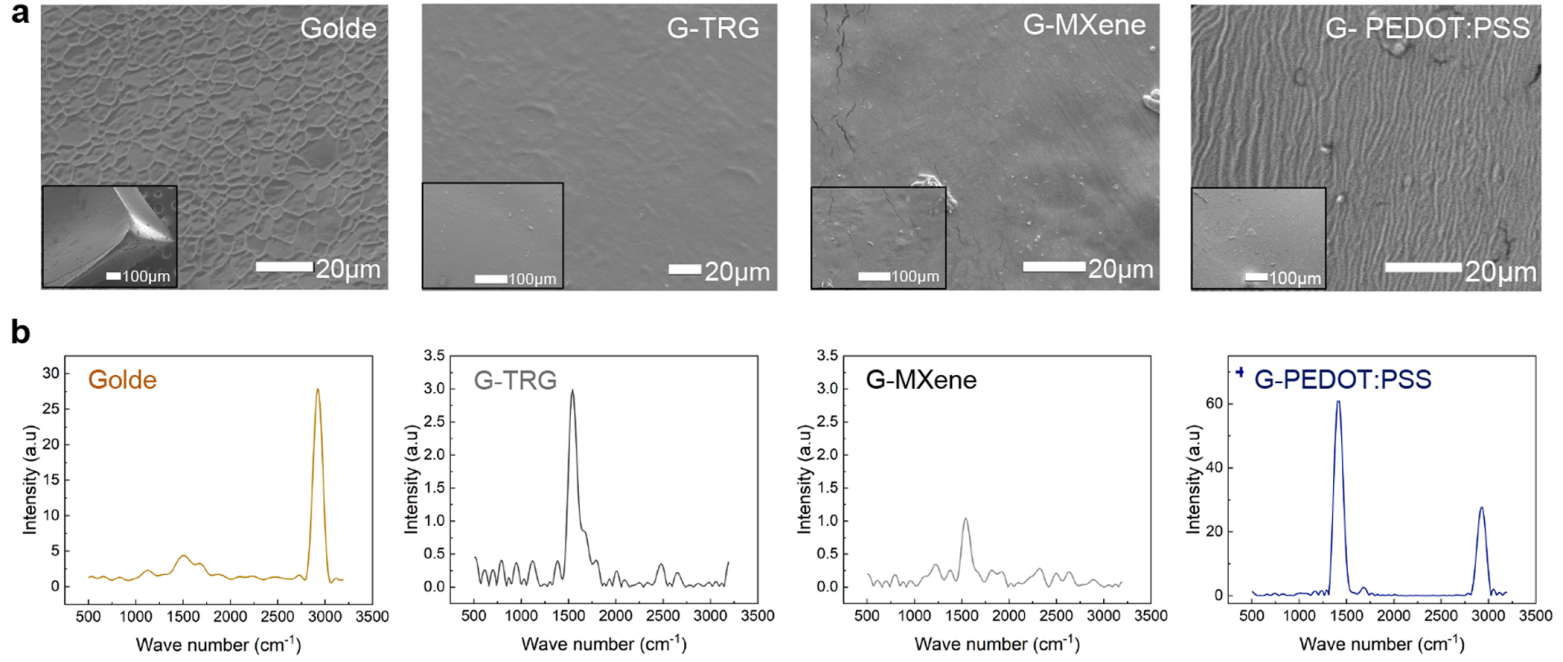
(a) SEM imaging and (b) Raman profiles of (from left to right) the base hydrogel *Golde* and the three conducting hydrogels G-TRG, G-MXene, and G-PEDOT:PSS.

The surface morphologies of the different hydrogels are shown in Figure 2a. *Golde* was found to have a grain-like surface morphology, most likely due to the amorphous architecture into which the biopolymers were intermixed. The addition of MXene and TRG resulted in a considerably smoother and uniform surface, which is desirable for realizing a conforming and skin-friendly electrode interface. This could be attributed to the fact that 2D carbon materials such as MXene and TRG restrict the polymer movement, thus inducing higher resistance before it elongates.^33,34^ Finally, the addition of PEDOT:PSS resulted in a wrinkly surface as a result of the interaction between the biopolymers and the conducting polymer shown previously. All scanning electron microscopy (SEM) images indicate the uniform integration of the conducting materials with the biopolymers, and the smoothness in all cases. This is expected to result in higher-quality ECG signals that are less affected by motion artifacts. X-ray diffraction (XRD) data are shown in Figure S2, which indicate that the *Golde* variants possess both a crystalline and an amorphous nature. Figure 2b shows the Raman spectra of the *Golde*, G-PEDOT: PSS, G-MXene, and G-TRG samples; for a more detailed Raman spectra analysis, refer to Figure S1. The *Golde* sample shows major Raman bands at 3356, 2929, and 1656 cm^–1^, which are attributed to O–H and N–H stretching, C–H stretching, C=O stretching (amide I), and NH bending, respectively. After blending with PEDOT:PSS, the major Raman bands of PEDOT are observed at 1521, 1448, 1381, and 1267 cm^–1^, which are assigned to the C_α_=C_β_ asymmetrical, C_α_=C_β_ symmetrical, C_β_=C_β_ stretching, and C_α_=C_α′_ inter-ring stretching vibrations, respectively. The vibrational modes of PSS are positioned at 1108 and 1003 cm^–1^. The G-MXene sample shows Raman bands over 500–750 cm^–1^, which are associated with vibrations of C atoms of Ti–C (E_1g_, E_2g_, and A_1g_ symmetries), and the G-TRG sample shows the characteristic D-band and G-band peaks of TRG in the composite at 1348 and 1518 cm^–1^, respectively. The observed characteristic peaks of MXene have low intensities, which possibly indicate that MXene is well dispersed in the *Golde* sample with no aggregations. The intensities of the D and G peaks are comparatively weak in the G-TRG sample, indicating that the exfoliated TRG layers are uniformly dispersed in *Golde*.

### Mechanical Characterization

**Figure 3:**
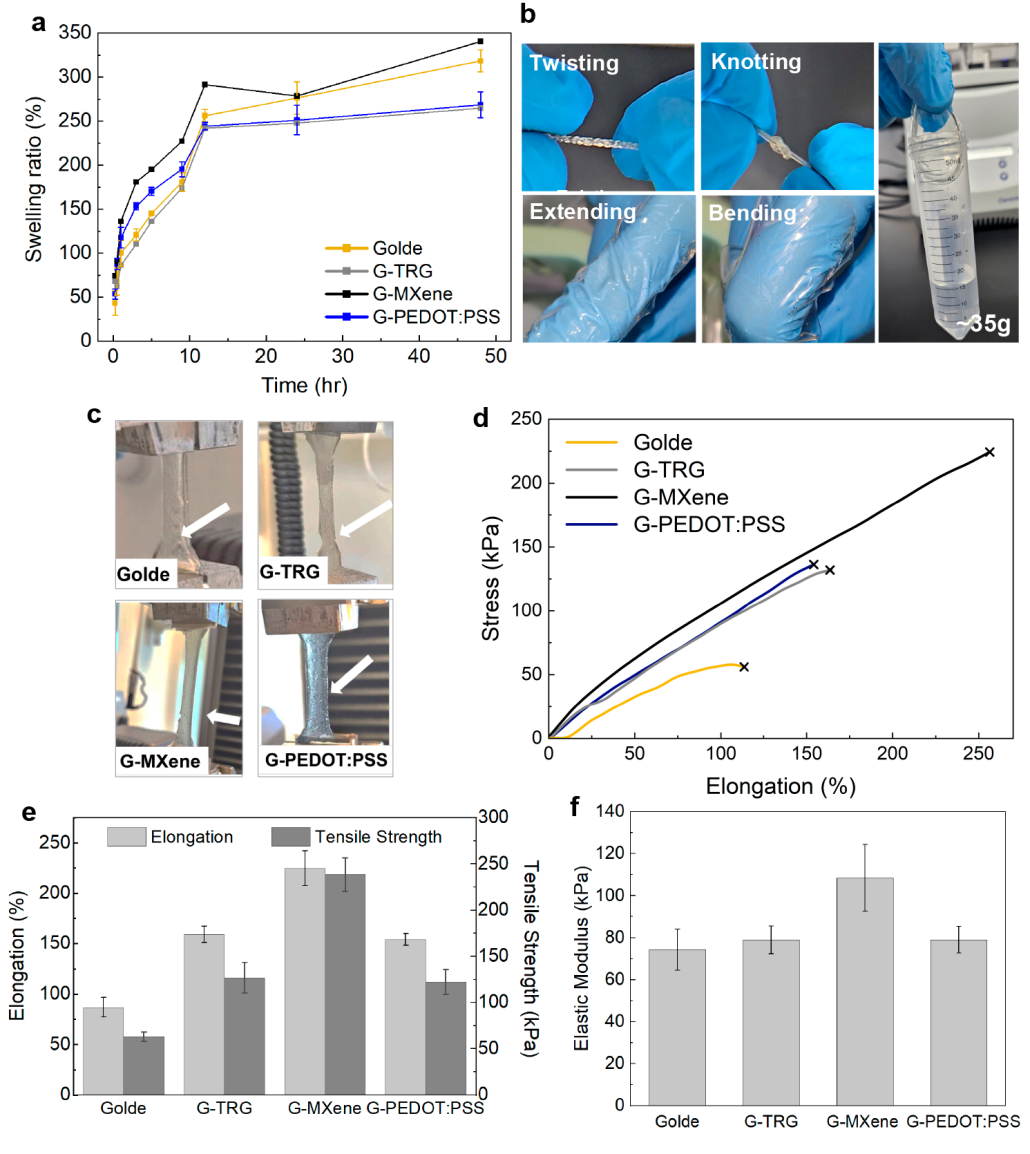
Mechanical characterization. (a) Swelling ratio profiles of the hydrogel variants. (b) Photographs of the hydrogel being twisted, knotted, stretched, and bent after 20 finger bending cycles, showing its conformability with gloves and its ability to carry ∼35 g of water when attached to a plastic container. (c) The dog-bone-shaped hydrogel samples in tensile testing setup (during failure). (d) Stress (kPa) vs elongation (%) curves; “×” indicates failure. (e) Tensile strength (kPa) and elongation (%) of each of the hydrogel electrode variants. (f) Comparative graph of the elastic modulus values among the hydrogel variants. All the above-mentioned plots were performed in triplets (*n* = 3).

Prior to testing the mechanical performance of the hydrogels, we performed a swelling test to evaluate the water uptake properties of the fabricated hydrogels, shown in Figure 3a. Initially, G-MXene and G-PEDOT:PSS experienced a similar trend with the highest swelling rate. After 12 h, G-TRG and G-PEDOT:PSS reached saturation, and by 48 h, all four hydrogels showed saturation. Figure 3b shows photographs of the hydrogel being twisted, knotted, stretched, and bent without these actions affecting its shape and robustness as well as its ability to hold a plastic tube with water weighing 35 g. To verify the mechanical properties of the fabricated hydrogels against human skin and other works in the literature, tensile testing was performed, as shown in Figure 3c, and a stress versus elongation curve was obtained for each variant, (Figure 3d). The tensile testing of the hydrogels showed that all the variants had an elastic modulus that mimics that of skin (0.5–500 kPa).^25^ Typically, a higher elastic modulus with stretchable behavior is desirable, as it minimizes electrode detachment or displacement during movement, thus enhancing stability, durability, and the signal-to-noise ratio. All variants demonstrated a relatively linear behavior which facilitated the estimation of their elastic moduli. As shown in Figure 3e,f, G-MXene was found to have the highest tensile strength of 239 kPa and an elastic modulus of 108kPa, followed by G-TRG (127 and 79 kPa), G-PEDOT:PSS (122and 79kPa), and finally *Golde* (63 and 74kPa). The superiority of G-MXene is mainly due to the ordered intercalated structure of MXene, which introduces more order into the hydrogel polymer matrix. Additionally, because of its high toughness and the abundance of functional groups present on its surface, it simultaneously restricts the movement of the hydrogel polymer chains, allowing them to tolerate more stress before fracture occurs in the material. Generally, the proposed conducting hydrogel electrodes outperform the state-of-the-art natural hydrogel electrodes proposed for electrophysiological applications, as summarized in Table S1.

### Electrochemical Characterization

**Figure 4:**
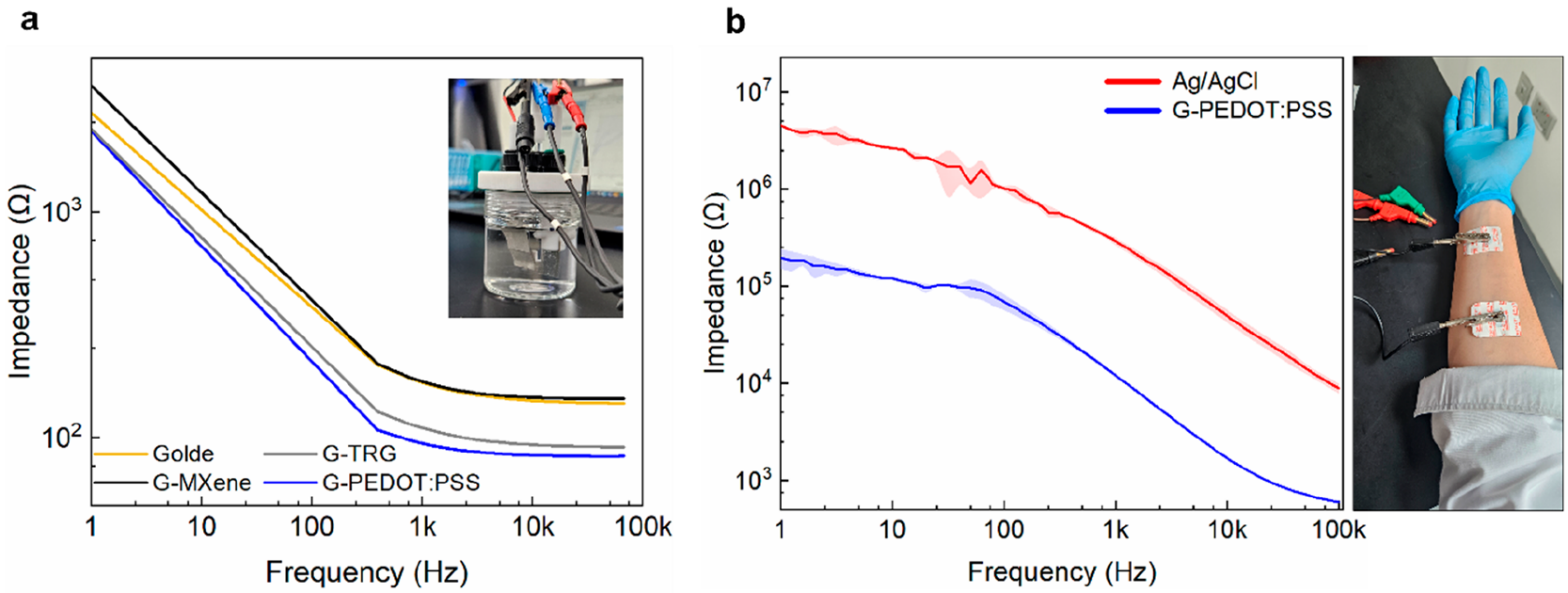
Electrical characterization. (a) Bode plot demonstrating the average frequency-dependent impedance response of the hydrogel electrode variants with an electrochemical cell setup at a frequency range of 1 Hz −100 kHz and using phosphate-buffered saline 1X with *n* = 3/variant. (b) Skin contact impedance setup as well as a plot comparing the commercial clinical-grade Ag/AgCl to G-PEDOT:PSS (lowest impedance variant) with *n* = 3.

Figure 4a shows a comparative Bode plot of the impedance of the four hydrogel variants over a wide range of frequencies from 1 Hz to 100 kHz. It should be noted that the curves shown in Figure 4a are the average curves of 3 samples/variant, where each was measured 3 times (i.e., 9 readings/variant). As expected, the impedance of the hydrogel electrodes demonstrated a clear transition from a resistive regime to a capacitive regime at lower frequencies due to the presence of a mixture of charge carriers^35^ (for more details, see Figure S3). Overall, the conducting hydrogels exhibited higher capacitance and lower resistance (hence, improved conductivity) compared to the base material, highlighting the uniform distribution of the conducting materials within the hydrogel base material and the favorable interactions with the (bio)polymeric network. The impedance values at the relevant frequencies for electrophysiological sensing are shown for easier comparison in Figure S3, (i.e., at 100 Hz, where a capacitive behavior is expected, and at 1 kHz, where the dominating impedance behavior is resistive). Similar to the work by Lee et al.,^36^ we also obtained skin contact impedance measurements, as shown in Figure 4b, directly comparing a commercial Ag/AgCl electrode to the G-PEDOT:PSS variant. A significant (more than 1 order of magnitude) decrease in the skin contact impedance was found in the case of the gel electrode. Overall, the low impedance of the hydrogel electrodes allows for efficient charge transfer between the electrodes and biological tissues, resulting in improved signal quality and reduced noise during electrophysiological recordings.

The electrochemical impedance spectroscopy (EIS) and cyclic voltammetry (CV) curves of the electrodes are shown in Figures S3 and S4, respectively. CVs were obtained at two different scan rates (0.1 and 0.05 mV/s) and in a potential range from −0.6 to 0.6 V. The CV analysis showed stable, reversible, and well-defined curves.^37^ In all samples, as the scan rate increased, the current and the area under the curve of the electrodes increased with little change in the shape of the CV, indicating effective ion transport processes. This effect was more pronounced with the G-TRG hydrogel electrode. The calculated specific capacitance area of each hydrogel variant is summarized in Figure S4, with G-PEDOT:PSS exhibiting the highest value, followed by G-TRG and G-MXene. The capacitive behavior of the hydrogel electrodes is thought to increase SNR,^7,8^ as they are not expected to undergo electrochemical reactions at the electrode–tissue interface that could interfere with accurate recordings.

The stability of the hydrogel electrodes (in terms of their electronic properties) was evaluated by monitoring the change in gain over frequency (Figure S5) for all four electrode variants after 42 days of storage in ambient room conditions. It can be seen that the electrode properties remain unchanged (see lines (fresh gels) versus symbols (old gels) in each electrode variant), highlighting their reusability (owing to their thermo-reversibility) and long-term storage capability. This is not possible with Ag/AgCl, which can neither be reused nor stored for long-term, as it tends to dry out and degrade. Overall, the results indicate that the hydrogel electrodes infused with conducting materials exhibit good electrical performance, superior to that of the base material alone, for the intended application with no apparent differences observed between the different variants.

### ECG Recordings: Commercial Ag/AgCl versus Conducting Hydrogel Electrodes

**Figure 5:**
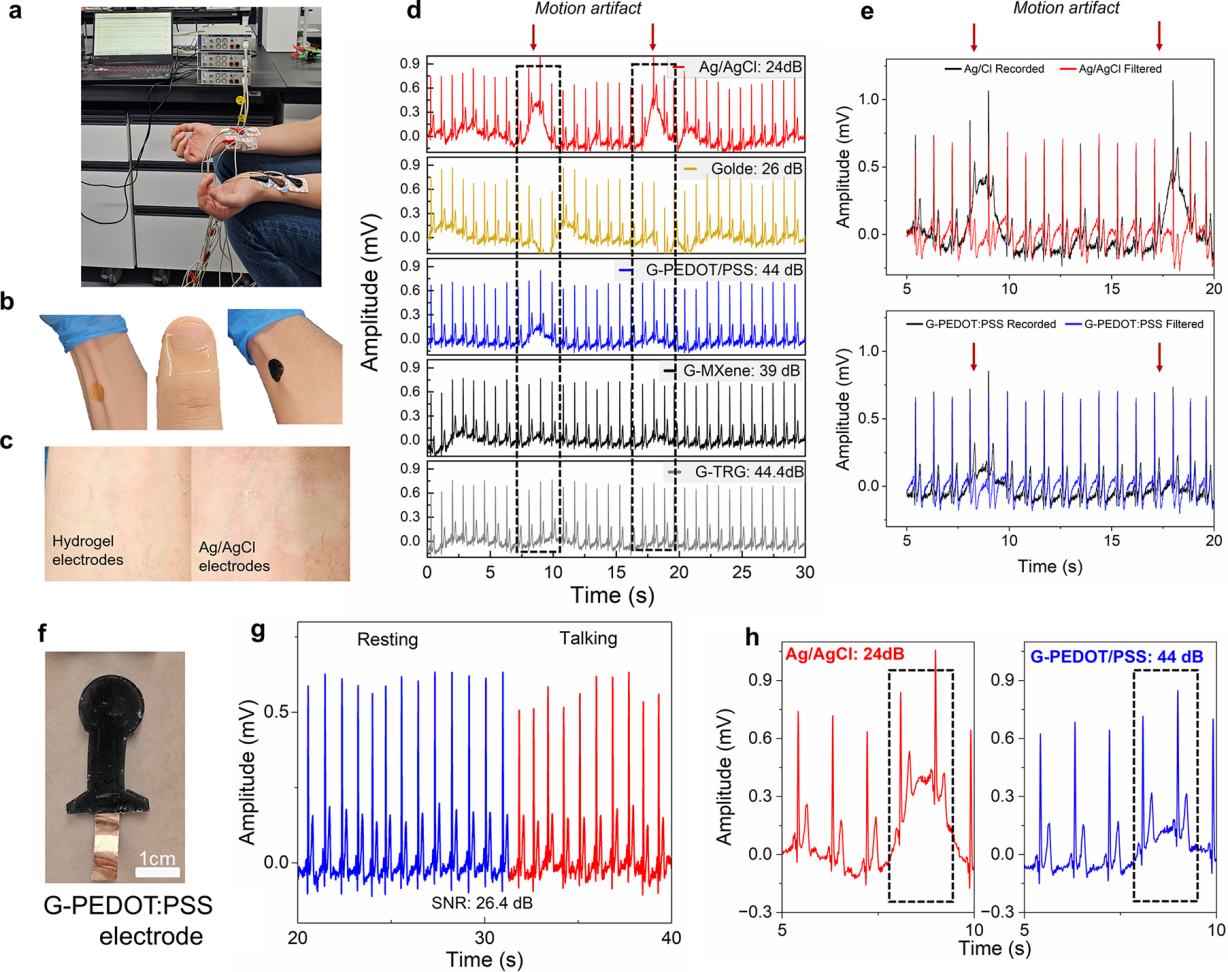
Electrophysiological recordings. (a) Photograph of ECG acquisition setup. (b) Photographs showing the conformability and stretchability of the self-adherent hydrogel variants. (c) Photographs of the skin after using the hydrogel electrodes for the 30 min ECG recording session compared to after using a conventional Ag/AgCl electrode. (d) Stacked ECG recording preview of all the electrodes, along with the corresponding signal-to-noise ratio (dB). (e) Effect of filtering the data for Ag/AgCl vs G-PEDOT:PSS. (f) G-PEDOT:PSS all-flexible electrode directly self-adhering to the skin using flexible copper tape for the interface. (g) ECG recording from the all-flexible electrode setup at rest and while talking. (h) The effect of a motion artifact on the recorded ECG signal on both the Ag/AgCl and G-PEDOT:PSS electrodes.

The low electrode impedance (Figure 4a), superior mechanical compliance (Figure 3 and Table S1), and inherent adhesiveness of the hydrogel electrodes demonstrates their suitability for acquiring ECG signals as efficient electrolyte-retention elements. Using a Lead I ECG placement, four sets of the same hydrogel electrodes and a set of Ag/AgCl electrodes were used to obtain the ECG signals from two consenting adult males. The ECG signals were recorded with a setup that was composed of a PowerLab instrument with a bioamplifier and a computer with LabChart software (Figure 5a). The hydrogel electrode variants were used separately for 1 min while at rest. The same electrodes were used for both volunteers. The resulting ECG signal features were visible, stable, and reproducible across the volunteers. Following this, all hydrogel electrodes and the commercial Ag/AgCl electrode were used simultaneously for 30 min to assess their performance during more extended recording periods. The G-hydrogels were attached to the hand of a representative participant (Figure 5a,b) in the same way (and at the same locations) as the commercial Ag/AgCl ECG electrodes for comparison. Remarkably, the attachment site of the hydrogel electrodes exhibited no apparent skin irritation following the 30 min ECG recording session. In contrast, in the case of the Ag/AgCl electrodes, we noted a slight skin redness and the occasional hair removal of the hair of the arms (Figure 5c), possibly due to the strong adhesiveness of the adhesive used. Moreover, the pH of the hydrogels was tested using litmus paper, and it was found to be in the range of 6–7 (Figure S7), which is in line with that of skin. According to the tensile data, the hydrogel electrodes had the same modulus range as skin, allowing for more than 30% elongation, which is the minimum amount required for over-the-skin hydrogel patches for wearable applications to conform and be unaffected by skin movement, as opposed to the rigid Ag/AgCl electrodes. Indeed, the G-hydrogels adhered well to the skin, possibly reducing contact imperfections caused by human activity (see their almost undisturbed ECG recordings compared to that of Ag/AgCl after a hand bending motion artifact, as indicated by arrows in Figure 5d).

Figure 5d illustrates a time domain representation of the ECG signals recorded simultaneously with the four hydrogel electrode variants and the commercial Ag/AgCl electrode. Distinguished and minimized fluctuating P, QRS, and T waves are clearly identified and are visible in the ECG waveforms for all the electrode variants. Additionally, the ECG signals from all of the electrode variants are very similar. The comparison of the SNRs of the signals acquired by the different electrodes offers a clear perspective on the relative performance of these electrodes. The SNR was calculated by extracting the signal power from the 0.5–40 Hz band and the noise power from 0–0.49 and 41–500 Hz. Superior signal clarity was demonstrated by the Ag/G-TRG electrode and Ag/G-PEDOT:PSS electrode with SNR values of ∼44.4 dB. The Ag/G-MXene electrode exhibited an SNR of 39 dB, while the Ag/*Golde* and Ag/AgCl electrodes exhibited the lowest values for SNR of 26 and 24 dB, respectively. Interestingly, our base hydrogel material exhibited a slightly higher SNR compared to the commercial electrode.

Figure 5e shows the effect of filtering the raw (as-recorded) data for a representative G-hydrogel electrode and a commercial one. The black data depict the raw ECG signals, while the colored data (red or blue) depict the signals after digital processing for Ag/AgCl and G-PEDOT:PSS using a MATLAB bandpass filter modified to a range of 0.5–40 Hz. In the G-PEDOT:PSS electrode recording, the R-peak values are almost identical after de-noising the signals, while the P, Q, S, and T waves differ only slightly, suggesting the superior ability of the hydrogel electrode to capture signals with minimal noise interference. This is also evidenced in the data recorded with a motion artifact, where, compared to Ag/AgCl, the hydrogels showed significantly reduced susceptibility to interference. Overall, the G-hydrogels acquired high-quality ECG signals after 30 min of continuous use with high SNRs and almost unaffected behavior during motion artifacts.

### Stability of the Hydrogels

The long-term stability of the electrodes was tested after three months of storage under ambient room conditions, and a decrease in the SNR by 18.2% was observed (Figure S6); however, they still outperformed the commercial electrode. Due to the hygroscopic nature of hydrogels, we also investigated the effect of humidity on the hydrogel electrode signal acquisition by enclosing the G-PEDOT:PSS electrodes in a room at the extreme condition of 95% relative humidity (RH) overnight, followed by a 10 min long ECG recording. The performance of the G-PEDOT:PSS hydrogel showed a 13.3% decrease in the SNR (see Figure S6). Lastly, the effect of sweat on the performance of the hydrogel was tested by performing ECG in sweat conditions; a negligible decrease of only 0.04% in the SNR was observed.

### All-Flexible Self-Adhesive Hydrogel for ECG

To further evaluate the “stand-alone” performance and skin-adhering properties of the hydrogel, the G-PEDOT:PSS electrode was fabricated as a proof of concept on an all-flexible setup using copper tape for the connection, as shown in Figure 5f, as a simpler, cheaper, and more eco- and skin-friendly solution to the commercially available one. The electrode design was based on a standard commercial clinical-grade Ag/AgCl electrode (Skintact) with a 1.75 cm diameter circle that would act as the active sensing area. Moreover, because the ECG signal direction is radially symmetric, a circular shape is preferred.^38^ A 1 cm × 1.3 cm rectangular area of the all-flexible electrode was used for interfacing the hydrogel with the copper tape. This area was covered and did not contribute to the signal acquisition (did not touch the skin). Since all G-variants exhibited similarly high performance, the choice of the G-PEDOT:PSS was based on its commercial availability, simplicity in processing, and low cost compared to MXene and TRG. We note that the hydrogel variant used here was stored for 42 days before rehydrating it and using it to develop the all-flexible electrode. Figure 5g shows the ECG recordings obtained using the all-flexible electrode on a consenting 24-year-old male at rest and while talking. The resulting ECG signal was clear, visible, and comparable to that of the commercial electrode. During resting or baseline conditions, the ECG signal exhibited a regular pattern with characteristic waves and intervals, while during slight activity (talking), we can see a change in the recorded ECG patterns (as expected) compared to baseline, possibly attributed to alterations in heart rate and autonomic nervous system activity. The flexible electrode was also found to be less susceptible to motion artifact interference compared to the commercial electrode (Figure 5h). Furthermore, its calculated SNR was found to be 26.4 dB (still higher than that of the Ag/AgCl and *Golde* electrodes), which was lower than that recorded for the same variant (∼44 dB) using the Ag conventional setup and adhesive G-PEDOT:PSS. We believe that this could be attributed to a number of factors related to the setup (e.g., copper tape) used to interface the electrode with the measurement unit, which necessitates further optimization, and/or to the adhesiveness of the hydrogel, which could also be improved. Nevertheless, the G-PEDOT:PSS flexible electrode was accurate in recording the ECG signal at rest and in identifying changes during slight activity (talking), exhibiting a slightly higher SNR than the commercial electrode. It was also found to be less susceptible to interference compared to the Ag/AgCl electrode. We note that a small improvement in the adhesion of the all-flexible hydrogel was observed after a small drop of a commercial vinegar (∼30 μL) was added, which was reflected in the SNR value that improving from 26.4 to 27.2 dB (see Figure S8). This could be attributed to an improved interaction between the hydrogel’s functional groups and the skin by lowering the pH.

## Conclusions

We have developed a self-adhering, reusable conducting hydrogel that does not require any crosslinker by using a simple one-pot synthesis method. The hydrogel is composed of a natural biopolymer blend (gelatin, chitosan, and glycerol) and is infused with either PEDOT:PSS, MXeneor TRG. The resulting hydrogel electrodes, namely *Golde* (biopolymer blend), G-PEDOT:PSS, G-MXene, and G-TRG, were characterized according to their structure, surface composition, as well as mechanical and electrical performance. The mechanical properties of the hydrogels match those of the skin (0.5–500 kPa)^25^ while exhibiting stretchability and robustness, outperforming similar natural hydrogels in literature. Morphological and structural analysis revealed favorable interactions between the conducting materials and the biopolymer network, which were further corroborated by the improvement in the mechanical and electrochemical properties of the conducting material-infused hydrogels compared to those of the base material, *Golde*. The hydrogel variants were used as an electrode alternative for the commercial Ag/AgCl used for electrophysiological recordings, in this case ECG readings. The recorded SNRs of the hydrogel electrodes were considerably higher than those of the commercial ones, and the hydrogel electrodes showed pain-free removal and no skin irritation after a 30 min ECG recording. Furthermore, benefiting from the inherent adhesiveness of the hydrogels, an all-flexible electrode setup was developed using copper tape for the connection. The ECG recordings showed less noise and motion artifact susceptibility compared to the commercially available Ag/AgCl. Moreover, although other variants might have shown better performance in some aspects, the G-PEDOT:PSS variant was overall the best when taking into account the cost and ease in processability, along with its SNR performance and skin friendliness. This simple and low-cost solution could be easily integrated into wearable setups, possibly enhanced with artificial intelligence (AI) for advanced health monitoring and analytics.^39^ Although herein demonstrated for ECG, we believe other types of measurements would result in similarly superior performance. Further, we believe that the presence of glycerol and the low sol–gel (glassy state) temperature (i.e., the thermo-reversibility of the hydrogel) provides a thixotropic nature, rendering it a potentially good candidate for extrusion-based hydrogel 3D printing, extending the application window of our platform.

## Materials and Methods

### Materials

Gelatin (bovine skin, Type B, G9391, U.S.), Chitosan (high molecular weight, deacetylated chitin, 419419, Ireland), acetic acid (100%), glycerol (99%), ethylene glycol (EG), 4-dodecylbenzenesulfonic acid (DBSA), and (3-glycidyloxypropyl)trimethoxysilane (GOPS) were obtained from Sigma-Aldrich. Aqueous PEDOT:PSS dispersion (Clevios PH 1000, 1 L, Germany) was obtained from Heraeus GmbH & Co. MAX phase (Ti_3_C_2_Al powder, ≤40 μm, Ukraine) was obtained from Carbon-Ukraine Ltd. Graphite powder (Sigma-Aldrich, 10 mesh), sulfuric acid (Sigma-Aldrich, ACS reagent, 95.0–98.0%), hydrochloric acid (Sigma-Aldrich, ACS reagent, 37%), lithium fluoride salt (Sigma-Aldrich), potassium permanganate (Fischer Scientific, C99%), and hydrogen peroxide (Sigma-Aldrich, 30 wt % in H_2_O) was used. Graphite oxide was prepared from natural graphite using an improved synthesis method proposed by Tour.

### Chitosan Solution Preparation

A chitosan solution of 0.5 wt % was prepared by measuring 10 mL of distilled water at 85 °C into a 50 mL borosilicate beaker, which was being stirred at 250 rpm. Then, 0.05 g of the chitosan powder was added, and 100 μL of acetic acid was added dropwise upon dispersion into the solvent. The solution was stirred for 2 h (until the chitosan dissolved completely). The solution should be transparent and thick without visible fibers.

### Preparation of the Hydrogel Solution

Following a simple one-pot process, 12 mL of distilled water was added to a 50 mL beaker and stirred at 160 rpm at a temperature no higher than 55 °C. In this order, 3 mL of glycerol was added, followed by 3 mL of the prepared chitosan solution. The mixture was left to mix thoroughly (not more than 5 min). Afterward, the stirring speed was slowly increased to 350 rpm, and 3.75 g of the gelatin powder was added slowly to avoid aggregation. After 20 min, the solution became homogeneous and transparent with a golden/amber color. To prepare larger amounts of the hydrogel solution, all of the ratios must be consistent.

### PEDOT:PSS Solution Preparation

First, 5% ethylene glycol (EG), 0.5% 4-dodecylbenzenesulfonic acid (DBSA), and 1% (3-glycidyloxypropyl)trimethoxysilane (GOPS) were added to the aqueous PEDOT:PSS dispersion. Using a digital scale in this order, EG, DBSA, and GOPS were added to a Falcon tube. After the addition of each additive, the solution was sonicated for 5 min (i.e., add EG, sonicate, add DBSA, etc.). Finally, a syringe filter of 0.8 μm pore size was used to complete the final mixture that would be used. GOPS was added to the PEDOT:PSS mix later (just before mixing it with the hydrogel solution) or not at all to avoid having clumps that would prevent the PEDOT:PSS from uniformly dispersing within the hydrogel solution.

### MXene Solution Preparation

The MAX phase substance, Ti_3_C_2_Al powder, with a size of up to 40 μm was utilized to produce MXene (Ti_3_C_2_T_*x*_) following a carefully refined minimally intensive layer delamination (MILD) process. This technique involved the targeted removal of aluminum through the in situ application of lithium fluoride salt (LiF), adopting a top-down strategy. In the initial step, the etching agent dissolved 3.2 g of LiF in 40 mL of 9 M HCl. Following this, 2 g of the MAX powder was gently introduced to the etching agent and agitated for 24 h at room temperature. Next, the MAX powder containing the etching agent was purified numerous times with DI water. This was accomplished by performing numerous centrifugation steps (each lasting 6 min at a speed of 3500 rpm) and sonication, with the slurry being discarded post-centrifugation in every wash cycle. This procedure concluded once the solution attained a pH higher than 6 and was a dark green colloidal suspension of singular or few-layered Ti_3_C_2_T_*x*_ flakes. The obtained MXene colloidal solution was then preserved in a freezer at −20 °C to maintain its stability for future use. A free-standing MXene film was produced using vacuum-assisted filtration to process the Ti_3_C_2_T_*x*_-rich colloidal solution through a filtration membrane (specifically, Celgard 3501 from Celgard, U.S.). After being air-dried for 24 h, the MXene (Ti_3_C_2_T_*x*_) film was carefully detached from the filtration membrane. The concentration of the MXene colloidal solution was determined by dividing the mass of the air-dried MXene film by the volume of the colloidal solution. Finally, dilution was performed to obtain an MXene colloidal solution concentration of 1 mg/mL using autoclaved double distilled water (18 MΩ).

### Synthesis of Graphene Oxide

TRG was synthesized in the lab. TRG is made by using graphene oxide as a raw material and thermally reducing it into a TRG powder.

Graphene oxide (GO) was synthesized using the Tour method: first, 300 mL of concd H_2_SO_4_ and 75 mL of orthophosphoric acid were mixed in a 500 mL beaker. Subsequently, 10.0 g of graphite powder was added to the above solution, and the mixture was stirred for 45 min at 30 °C. Then, KMnO_4_ (58.0 g) was slowly added to the mixture. The mixture was stirred at 25–30 °C for 72 h. Then, 150 mL of a 5% H_2_O_2_ solution was added to the mixture, and it was stirred for 2 h at 30 °C. The content was allowed to settle before it was centrifuged to remove the superannuated solution. The residue was washed with 1 M HCl followed with DI water. The residue was further purified using a cellulose membrane for 1 week in DI water medium. The purified residue was dried under a freeze drier, and graphite oxide (GO) was obtained as a solid powder.

### Synthesis of Thermally Reduced Graphene (TRG) Solution

Thermally reduced graphene (TRG) was prepared by placing GO (300 mg) into a 1.3 m long quartz tube (inner diameter of 25 mm). The opening end of the quartz tube was closed by using a rubber stopper. A nitrogen inlet was then inserted through the rubber stopper. The sample was flushed with nitrogen for 15 min, and the quartz tube was quickly inserted into a tube furnace that was pre-heated to 1000 °C, and it was held in the furnace for 30 s.

### Conducting Hydrogel Samples

*Golde* hydrogel solution in the sol-state was placed on a hot-plate stirrer at 55 °C and stirred at 500 rpm. At a ratio of 10% (v/v), 0.5 mL of the conducting material solution was added dropwise into 5 mL of the *Golde* solution. The resulting solution was stirred for 10 min. At the same temperature, the stirring speed was lowered to 90 rpm, and the solution was stirred for 10 min to move any bubbles to the surface.

### All-Flexible Electrode Fabrication

A 3D mold of the flexible electrode was designed using TinkerCAD and 3D-printed into a flexible polyurethane mold using an ELEGOO Mars 3 Pro 3D printer. The conducting hydrogel electrode was attached to copper tape that had a metal stud to connect to the ADInstruments PowerLab 26T bioamplifier with only the head (active site) of the flexible electrode exposed to the skin, while the tail was covered to achieve a comparable result to previously tested electrodes; furthermore, in future tests and real-life applications, the setup would be further improved, and the exposed hydrogel area would be maximized to include the entire hydrogel area, thereby improving the signal quality. The ADInstruments LabChart application was used to record and analyze the Lead I ECG signal.

### Characterization

#### Morphological

To characterize the hydrogel surface morphology, SEM imaging was conducted. To assess the available functional groups found throughout the hydrogel, Raman spectroscopy was used instead of Fourier transform infrared (FTIR) spectroscopy because it is less sensitive to perturbations caused by the water molecules present in the hydrogel samples. Finally, since the material organization (i.e., amorphous, semicrystalline) in these hydrogel composite electrodes affects their surface morphology and thus their interactions with the skin (e.g., wettability and contact area) and their mechanical properties, XRD analysis was performed to improve our understanding of the material structure.

#### Sample Preparation for Analysis

In the sol-state, the hydrogel samples were cast into a 3D-printed mold that was 6 mm in diameter and 1.3 mm thick. Next, the hydrogels were placed in a −20 °C freezer for 2 h. Then, the frozen samples were rapidly moved into a freeze drier and placed there for 24 h (until the samples were completely freeze-dried). Finally, the sample surfaces were gold-coated to make them visible in the SEM scan. To perform SEM imaging, a JEOL JSM-7610F SEM was used.

#### Mechanical Properties

##### Hydrogel Sample Preparation

As distilled water was the main solvent of the hydrogels, all hydrogel samples were cast into a 3 cm × 2 cm mold and soft-baked at 58 °C for around 10 min (an estimate based on observations); this was done to reduce the amount of free water in the hydrogel matrix, thus stabilizing it. This specific temperature is not mandatory; however, we observed that at lower temperatures, the hydrogels took a long time to become ready. At higher temperatures, the hydrogels lost water rapidly (less controlled), and we believe higher temperatures could cause some of the conducting materials to start degrading. Next, the hydrogels were left to cool on a tabletop for 5 min. The tensile testing of our hydrogels was performed under ambient room conditions to simulate the conditions that they would be exposed to during their application.

##### Tensile Testing

Using an Instron 5966 universal testing machine with a 10 kN load cell, an extension rate of 0.30 mm/s, and a 5 N maximum load, the hydrogels were fixed with a clamp sample holder and tested. A small piece of tissue paper and duct tape were used to firmly attach the sample to the clamp without destroying the samples. The dog-bone-shaped samples were stretched to their breaking point while their stress/strain response was plotted.

#### Electrical Properties

##### Electrochemical Impedance Spectroscopy (EIS)

EIS was carried out using an electrochemical impedance analyzer (AutoLab PGSTAT204, Sweden) with a sine wave with an amplitude of 10 mV and a frequency ranging from 0.1 Hz to 100 kHz with a focus window of 1 Hz to 100 kHz. The hydrogel sample had the dimensions 16 mm × 14 mm and a thickness of 1.5 mm.

##### Cyclic Voltammetry (CV)

CV was performed on the same hydrogel samples at scan rates of 0.05 and 0.1 V/s and in the potential window from −0.6 to 0.6 V. Specific capacitance values were calculated from the CV data.

##### Gain Response Setup

The gain response versus frequency plots for both the commercial and hydrogel electrodes were obtained using National Instrument tools, namely, the NI ELVIS II+ board. The experimental protocol employed a sinusoidal signal with a peak amplitude 0.1 V, and a frequency sweep ranging from 1 Hz to 100 kHz was used as the stimulus.

##### Swelling Ratio

The hydrogels were placed in a Petri dish under ambient room conditions with an adequate amount of deionized water to submerge the hydrogels. Afterward, the change in their weights was recorded using a balance, and finally, the swelling ratio was estimated; this was performed in triplets (*n* = 3) per variant over the course of 0.5, 1, 3, 5, 9, 12, 24, and 48 h.

##### Chicken Skin Preparation for Adhesive Testing

First, the chicken thighs were deskinned. Then, the skin was washed with 70% ethanol, dried off, and placed on a piece of aluminum foil.

## Supporting Information Available

Demonstration of the transparency, self-adherence, stretchability, and robustness of the *Golde* hydrogel (MP4)
Raman spectra, X-ray diffraction analysis, electrochemical impedance spectra, comparative impedance values at frequencies of interest (100 Hz and 1 kHz), cyclic voltammetry curves, specific capacitance values, gain response, hydrogel stability (effect of storage humidity levels and human sweat on the SNR), litmus paper pH test of the *Golde* hydrogel, effect of vinegar on the SNR of the all-flexible electrode, and a table benchmarking our work with similar ones in the literature (PDF)
