# Supplementary material for: One-Pot Synthesis of a Robust Crosslinker-Free Thermo-Reversible Conducting Hydrogel Electrode for Epidermal Electronics
Source: ACS Appl Mater Interfaces. 2024 Jan 12;16(45):61435–45. doi: 10.1021/acsami.3c10663 (PMC11565476; doi:10.1021/acsami.3c10663)
Supplement: Supplementary file 2 — am3c10663_si_002.pdf [file am3c10663_si_002.pdf]

Supporting Information

One-pot synthesis of a robust crosslinker-free thermo-reversible conducting hydrogel electrode for epidermal electronics

Nazmi B. Alsaafeen[a,d], *Sarah S. Bawazir* [a.] , *Kishore K. Jena*[a] , *Aibobek Seitak*[a], *Bushara Fatma*[b] *Charalampos Pitsalidis*[b,c], *Ahsan Khandoker*[a,c] , *Anna-Maria Pappa*[a,c,d]

a) Department of Biomedical Engineering, Khalifa University, Abu Dhabi, UAE

b) Department of Physics, Khalifa University, Abu Dhabi, UAE

c) Healthcare Engineering Innovation Center, Khalifa University, Abu Dhabi, UAE

d) Center for Separations and Catalysis, Khalifa University, Abu Dhabi, UAE

Email of corresponding authors: charalampos.pitsalidis@ku.ac.ae ; ahsan.khandoker@ku.ac.ae; anna.pappa@ku.ac.ae

Raman Spectroscopy

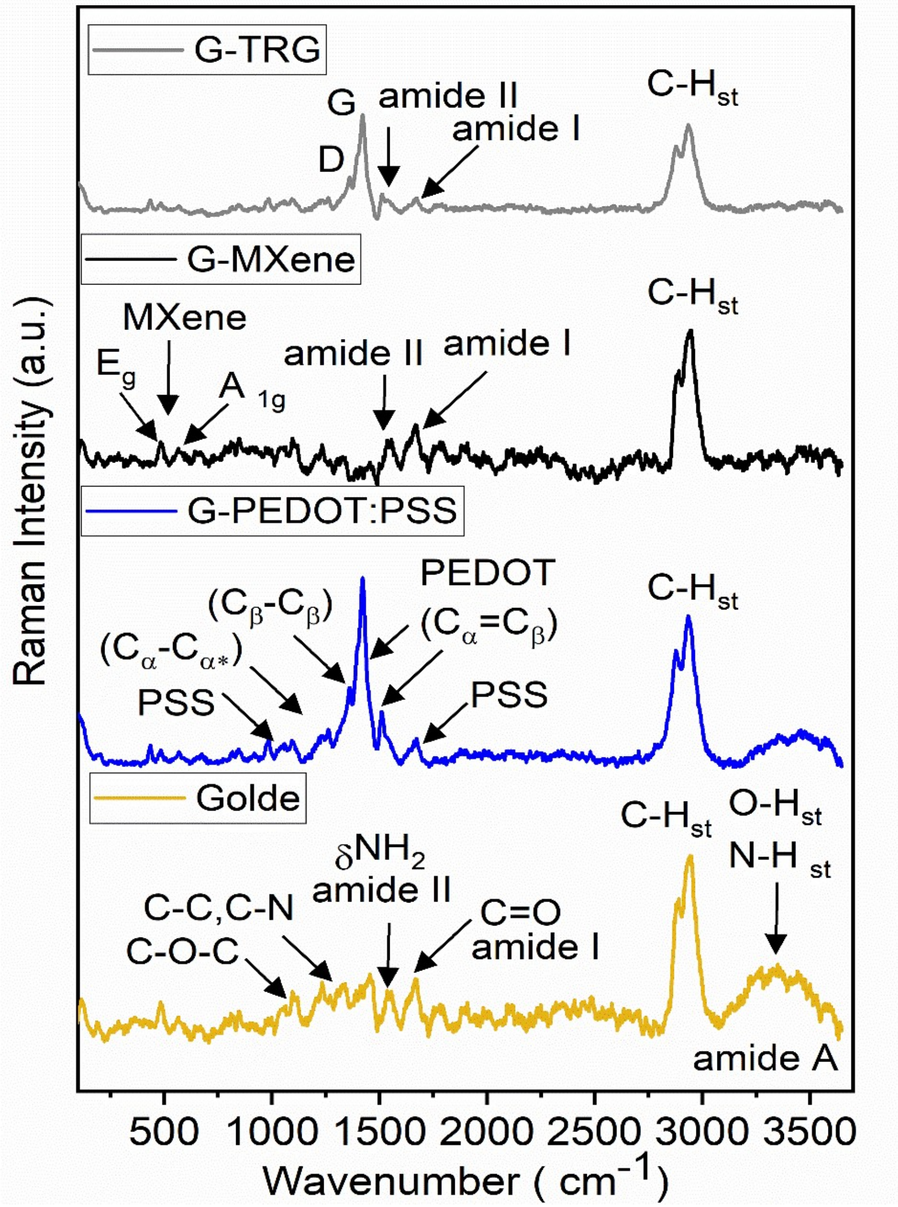

Figure S1: Full Raman spectra of Golde, G-PEDOT: PSS, G- MXene and G-TRG samples [13-16].

XRD analysis

As shown by figure S2, the XRD patterns of Golde, G-PEDOT: PSS, G-MXene and G-TRG. The sharp peak and broad peak for Golde sample observe at 2θ = 7.7° and at 2θ= 22.45°. This indicates Golde sample possess both crystalline and amorphous nature. After blend with PEDOT: PSS, the G-PEDOT: PSS sample shows one peak at 2θ = 8.8° but the broad peak at 2θ= 22.45° from Golde completely disappeared. This could be due to the better compatibility between Golde and PEDOT: PSS, which increases the amorphous nature of the material. In G-MXene and G-TRG samples the board peaks at 2θ= 23.55° and 2θ =21.75° and sharp peaks at 2θ = 8.01° and 2θ = 6.99°, respectively are again observed. This could be due to the regular sutural arrangement of Golde samples after the addition of MXene and TRG. This regular sutural arrangement may be happened due to the nucleating agent effect MXene and TRG in the hybrid materials. [1,2]

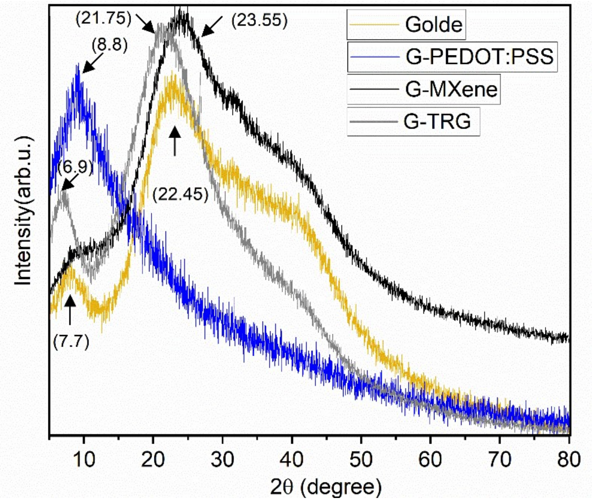

Figure S2: XRD profile of Golde, G-PEDOT: PSS, G-Mxene and G-TRG samples.

Electrical Characterization

As shown in equation 1, the reactance is inversely proportional to the frequency (f) of the current passing through the material with capacitance (C). Another contributing element is the frequency independent real element (resistance, R) which is a scalar. As shown by equation 2, the total impedance (Ztotal) is the vector sum of its resistance and reactance, as frequency decreases the reactance contribution becomes the dominating element.[34] Therefore at the highest frequencies the impedance is stable.

$$Equation\ 1. \qquad Xc = \frac{1}{2\pi f C}$$

$$Equation\ 2. \qquad Z_{total} = R + jX_c$$

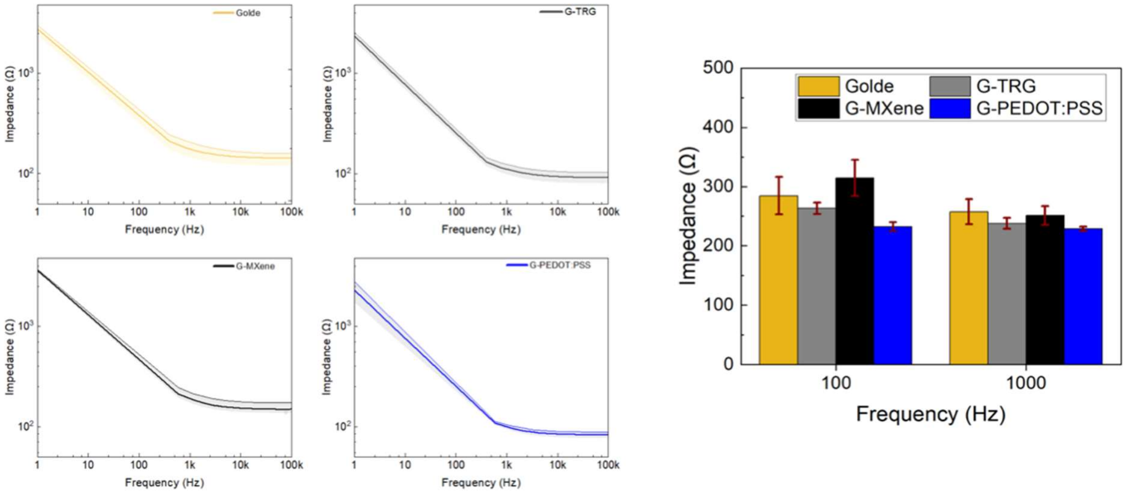

Figure S3: The electrochemical impedance spectroscopy of each hydrogel variant (n=6) and comparative impedance values at the relevant 100 and 1000 frequencies.

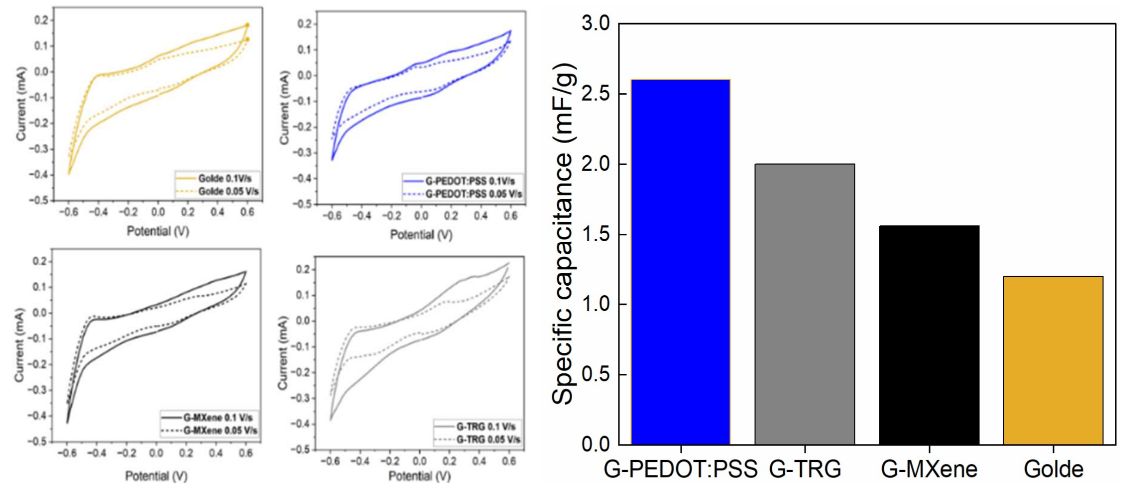

Figure S4: CV characterization (after 3 cycles) and calculated specific capacitance for each hydrogel variant.

Stability measurements

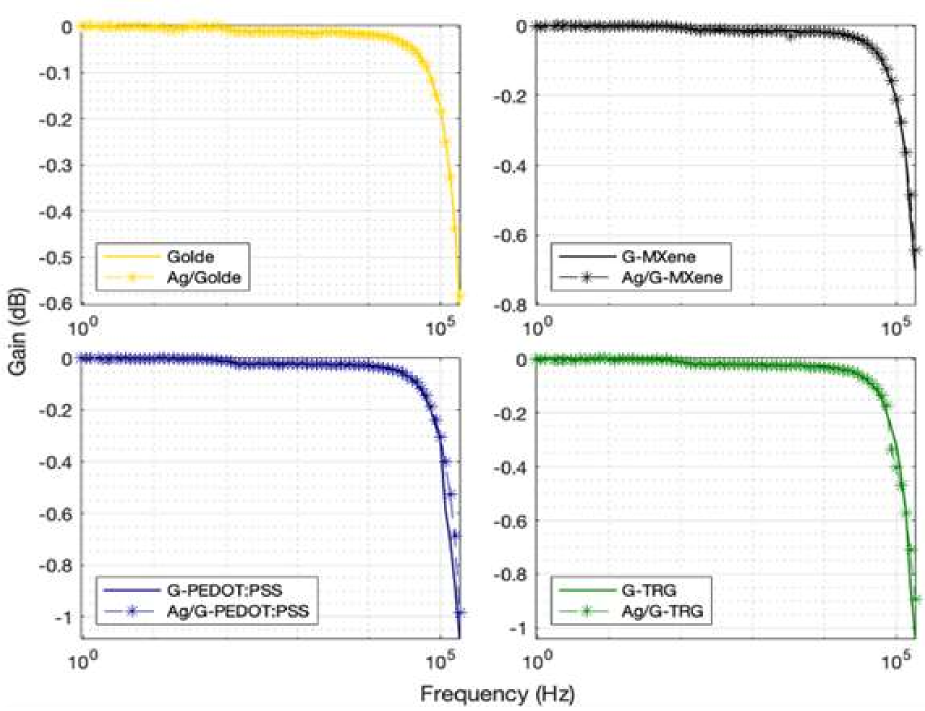

Figure S5: Gain response stability from 1Hz -10kHz of the fresh standalone hydrogel electrodes compared to electrode samples stored 42 days (see line vs symbols).

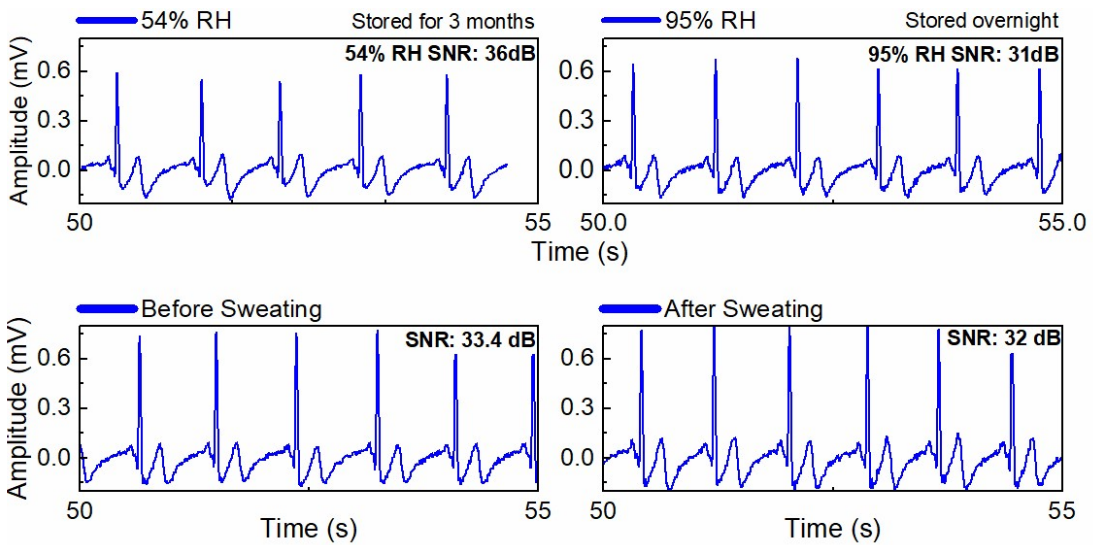

Figure S6: The effect of storing in different humidity levels and sweat on the signal to noise of the ECG recordings.

pH of the hydrogel (Sol state)

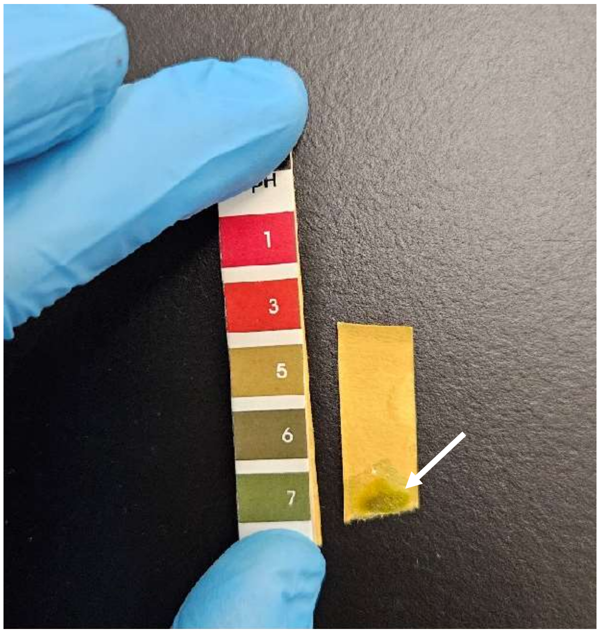

Figure S7: Litmus paper test of *Golde* hydrogel

All Flexible electrode ECG with and without vinegar coating

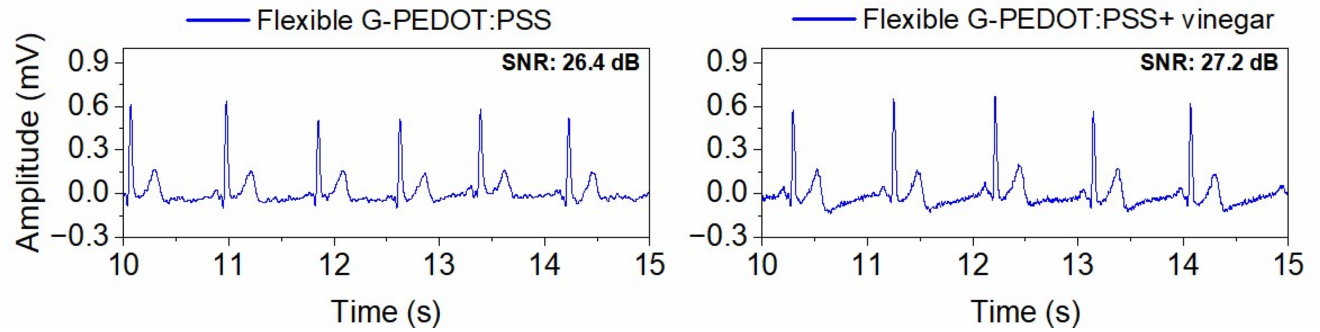

Figure S8: Effect of vinegar coating on all-flexible electrode.

Table S1 A comparison of the hydrogel electrodes in this work and other recent relevant works for electrophysiological acquisition in terms of tensile strength and elongation at break with some using chemical or physical cross-linking agent. Other works in the literature are either not tested using the same method or not designed for our application. All materials abbreviations are shown below.

Material	Base Material	Tensile Strength (kPa)	Elongation At break (%)	References
PVA, PPy and aramid nanofibers	Synthetic	9400	36	[3]
Polymerizable rotaxane hydrogel (PR-Gel)	Synthetic	78	830	[4]
PAAm/AAc/Glycerol	Synthetic	62	500	[5]
PNIPAM/AAc/Am/LMA	Synthetic	1010	1866	[6]
PVA/PAAm/LS/MXene	Synthetic	630	2000	[7]
PVA/CMC /PEDOT:PSS	Hybrid	85	74	[8]
PAA/ACC/MXene	Hybrid	-	450	[9]
Gel/PPy/rGO	Hybrid	63	250	[10]
AgNPs/MXene/GG/Alg-PBA	Hybrid	25	167	[11]
Gel/PEDOT:PSS/Genipin	Natural	72	235	[12]
Gel/CS/Glycerol	Natural	63	87	This work
G-PEDOT:PSS	Natural	122	154	This work
G-MXene	Natural	239	225	This work
G-TRG	Natural	127	159	This work

Abbreviations: Poly-vinyl alcohol (PVA), Polypyrrole(Ppy), reduced graphene oxide (rGO), Acrylic acid (AA), (PNIPAM), Acrylamide (Am), Polyacrylamide (PAAm), lauryl methacrylate (LMA), Lithium salt (LS), Thermally reduced graphene (TRG), N,N′-methylenediacrylamide (MBA, MBAm), Guar gum (GG) and phenylboronic acid grafted sodium alginate (Alg-PBA), Gelatin (Gel), Chitosan (CS), silver nanoparticles (AgNPs), Carboxymethyl cellulose (CMC), polyethylene-3,4-dioxythiophene:sodium polystyrene sulfonate (PEDOT:PSS).

REFERENCES

[1] Salem, K. S.; Lubna, M. M.; Rahman, A. F. M. M.; NurNabi, M.; Islam, R.; Khan, M. A. The Effect of Multiwall Carbon Nanotube Additions on the Thermo-Mechanical, Electrical, and Morphological Properties of Gelatin-Polyvinyl Alcohol Blend Nanocomposite. J. Compos. Mater. 2015, 49 (11), 1379–1391.

[2] Minus, M. L.; Chae, H. G.; Kumar, S. Interfacial Crystallization in Gel-Spun Poly(Vinyl Alcohol)/Single-Wall Carbon Nanotube Composite Fibers. Macromol. Chem. Phys. 2009, 210 (21), 1799–1808.

[3] He, H.; Li, H.; Pu, A.; Li, W.; Ban, K.; Xu, L. Hybrid Assembly of Polymeric Nanofiber Network for Robust and Electronically Conductive Hydrogels. *Nat Commun* **2023**, *14* (1), 759. https://doi.org/10.1038/s41467-023-36438-8.

[4] Xiong, X.; Chen, Y.; Wang, Z.; Liu, H.; Le, M.; Lin, C.; Wu, G.; Wang, L.; Shi, X.; Jia, Y.-G.; Zhao, Y. Polymerizable Rotaxane Hydrogels for Three-Dimensional Printing Fabrication of Wearable Sensors. *Nat Commun* **2023**, *14* (1), 1331. https://doi.org/10.1038/s41467-023-36920-3.

[5] (Carvalho, F. M.; Lopes, P.; Carneiro, M.; Serra, A.; Coelho, J.; de Almeida, A. T.; Tavakoli, M. Nondrying, Sticky Hydrogels for the Next Generation of High-Resolution Conformable Bioelectronics. *ACS Appl Electron Mater* **2020**, *2* (10), 3390–3401. https://doi.org/10.1021/acsaelm.0c00653.

[6] Liang, Q.; Xia, X.; Sun, X.; Yu, D.; Huang, X.; Han, G.; Mugo, S. M.; Chen, W.; Zhang, Q. Highly Stretchable Hydrogels as Wearable and Implantable Sensors for Recording Physiological and Brain Neural Signals. *Advanced Science* **2022**, *9* (16), 2201059. https://doi.org/10.1002/advs.202201059.

[7] Li, Q.; Zhi, X.; Xia, Y.; Han, S.; Guo, W.; Li, M.; Wang, X. Ultrastretchable High-Conductivity MXene-Based Organohydrogels for Human Health Monitoring and Machine-Learning-Assisted Recognition. *ACS Appl Mater Interfaces* **2023**, *15* (15), 19435–19446. https://doi.org/10.1021/acsami.3c00432.

[8] Wang, Y.; Qu, Z.; Wang, W.; Yu, D. PVA/CMC/PEDOT:PSS Mixture Hydrogels with High Response and Low Impedance Electronic Signals for ECG Monitoring. *Colloids Surf B Biointerfaces* **2021**, *208*, 112088. https://doi.org/10.1016/j.colsurfb.2021.112088.

[9] Li, X.; He, L.; Li, Y.; Chao, M.; Li, M.; Wan, P.; Zhang, L. Healable, Degradable, and Conductive MXene Nanocomposite Hydrogel for Multifunctional Epidermal Sensors. *ACS Nano* **2021**, *15* (4), 7765–7773. https://doi.org/10.1021/acsnano.1c01751.

[10] Yang, X.; Cao, L.; Wang, J.; Chen, L. Sandwich-like Polypyrrole/Reduced Graphene Oxide Nanosheets Integrated Gelatin Hydrogel as Mechanically and Thermally Sensitive Skinlike Bioelectronics. *ACS Sustain Chem Eng* **2020**, acssuschemeng.0c01998. https://doi.org/10.1021/acssuschemeng.0c01998.

[11] Li, M.; Zhang, Y.; Lian, L.; Liu, K.; Lu, M.; Chen, Y.; Zhang, L.; Zhang, X.; Wan, P. Flexible Accelerated-Wound-Healing Antibacterial MXene-Based Epidermic Sensor for Intelligent Wearable Human-Machine Interaction. *Adv Funct Mater* **2022**, *32* (47), 2208141. https://doi.org/10.1002/adfm.202208141.

[12] Lee, Y.; Yim, S.-G.; Lee, G. W.; Kim, S.; Kim, H. S.; Hwang, D. Y.; An, B.-S.; Lee, J. H.; Seo, S.; Yang, S. Y. Self-Adherent Biodegradable Gelatin-Based Hydrogel Electrodes for Electrocardiography Monitoring. *Sensors* **2020**, *20* (20), 5737. https://doi.org/10.3390/s20205737.

[13] Garreau, S.; Duvail, J. L.; Louarn, G. Spectroelectrochemical Studies of Poly(3,4-Ethylenedioxythiophene) in Aqueous Medium. Synth Met 2001, 125 (3), 325–329. https://doi.org/10.1016/S0379-6779(01)00397-6.

[14] Han, Y.-K.; Chang, M.-Y.; Huang, W.-Y.; Pan, H.-Y.; Ho, K.-S.; Hsieh, T.-H.; Pan, S.-Y. Improved Performance of Polymer Solar Cells Featuring One-Dimensional PEDOT Nanorods in a Modified Buffer Layer. J Electrochem Soc 2011, 158 (3), K88. https://doi.org/10.1149/1.3534201.

[15] Sarycheva, A.; Makaryan, T.; Maleski, K.; Satheeshkumar, E.; Melikyan, A.; Minassian, H.; Yoshimura, M.; Gogotsi, Y. Two-Dimensional Titanium Carbide (MXene) as Surface-Enhanced Raman Scattering Substrate. The Journal of Physical Chemistry C 2017, 121 (36), 19983–19988. https://doi.org/10.1021/acs.jpcc.7b08180.

[16] Jena, K. K.; Mayyas, A. T.; Mohanty, B.; Jena, B. K.; Jos, J. R.; AlFantazi, A.; Chakraborty, B.; Almarzooqi, A. A. Recycling of Electrode Materials from Spent Lithium-Ion Batteries to Develop Graphene Nanosheets and Graphene–Molybdenum Disulfide Nanohybrid: Environmental Benefits, Analysis of Supercapacitor
